# Supplementary material for: Observed interannual changes beneath Filchner-Ronne Ice Shelf linked to large-scale atmospheric circulation
Source: Nat Commun. 2021 May 20;12:2961. doi: 10.1038/s41467-021-23131-x (PMC8138005; doi:10.1038/s41467-021-23131-x)
Supplement: Supplementary file 1 — Supplementary Information [file 41467_2021_23131_MOESM1_ESM.pdf]

Supplementary Material for:

Observed interannual changes beneath Filchner-Ronne Ice Shelf linked to large-scale atmospheric circulation

Authors: Tore Hattermann[1,2,3*] Keith W. Nicholls[4], Hartmut H. Hellmer[1], Peter E. D. Davis[4,] Markus Janout[1], Svein Østerhus[5] , Elisabeth Schlosser[6,7], Gerd Rohardt[1], Torsten Kanzow[1,8]

Affiliations:

[1]Alfred-Wegener-Institut AWI, Helmholz-Zentrum für Polar- und Meeresforschung, Bremerhaven, Germany

[2]Akvaplan-niva AS, Tromsø, Norway

[3]Norwegian Polar Institute, Tromsø, Norway

[4] British Antarctic Survey BAS, Cambridge, United Kingdom

[5] NORCE and BCCR, Bergen, Norway

[6] Department of Atmospheric and Cryospheric Sciences, University of Innsbruck, Austria

[7] Austrian Polar Research Institute, Vienna, Austria

[8] Bremen University, Department of Physics and Electrical Engineering, Bremen, Germany

[*]corresponding author; tore.hattermann@npolar.no

Content:

Supplementary Figure 1

Supplementary Figure 2

Supplementary Table 1

Supplementary Table 2

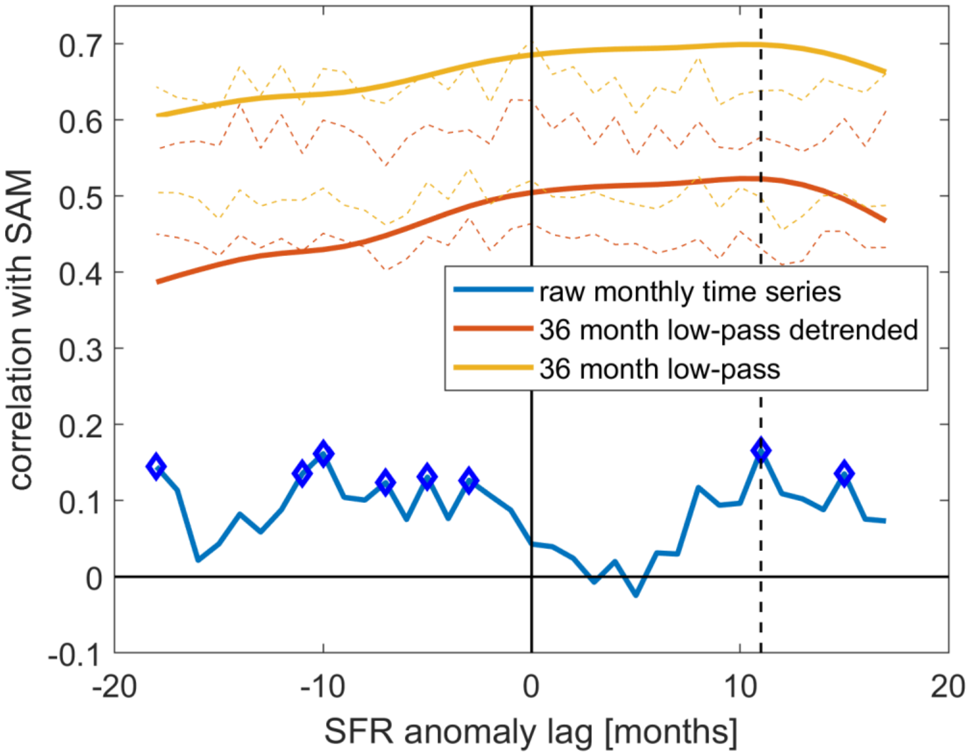

Supplementary Figure 1. **Time-lagged correlation coefficients between SFR anomalies and SAM-index.** Positive *x*-values indicate lag of SFRs behind SAM. Blue daimonds indicate statistically significant (p=.01) correlations of the monthly time series. Dashed lines indicate 95 % and 99 % significance levels from the Monte Carlo bootstrapping method for correlations of the low-pass filtered time series at corresponding color. I.e. most correlations of the low-pass

filtered series are significant above 99 % confidence for positive lag, whereas significance of correlations for the low-pass filtered and de-trended time series ranges between 95 % 99 %.

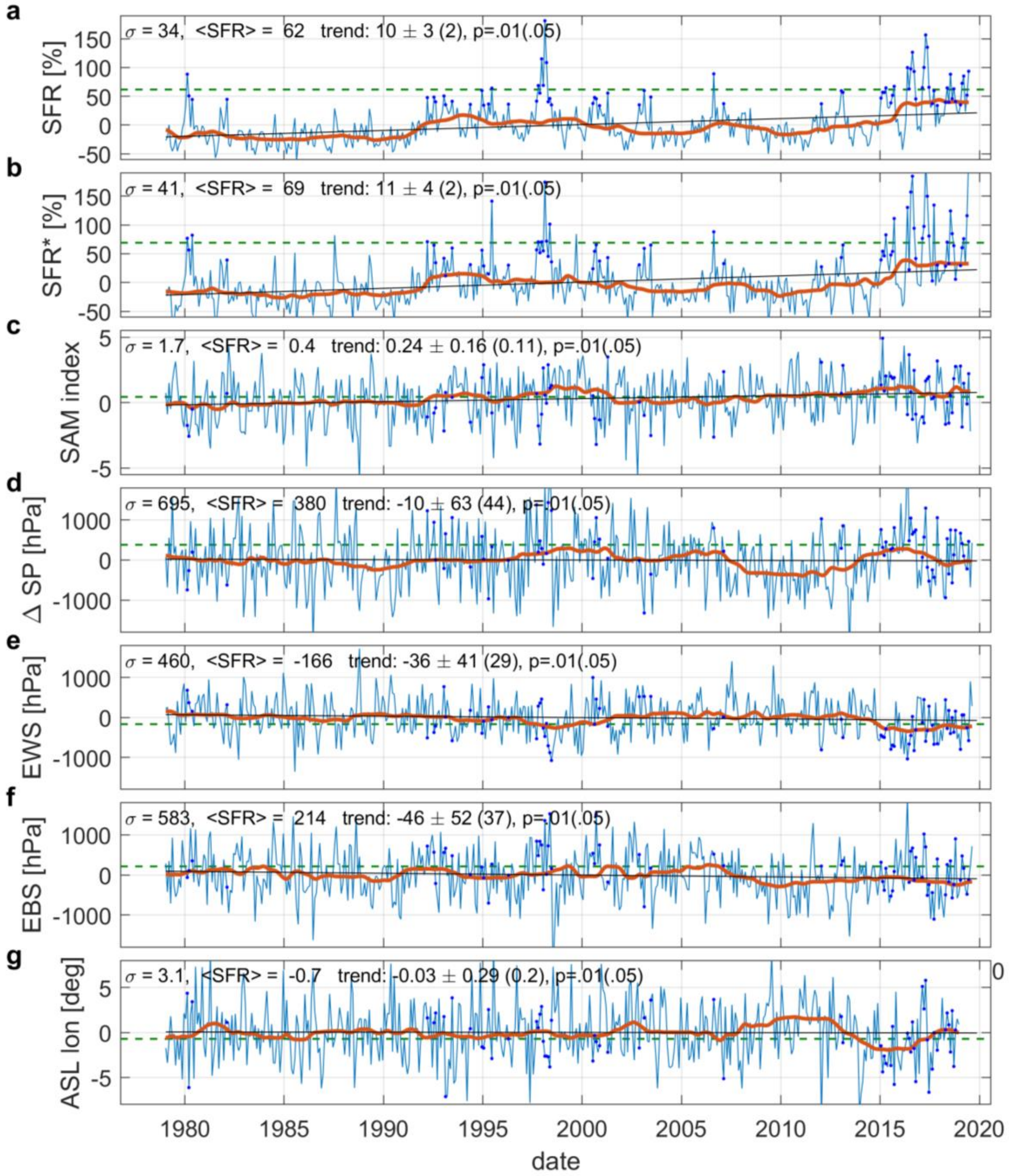

Supplementary Figure 2. **Time series of climatic indices.** Monthly and 36-month low-pass filtered time series of **a** sea ice formation rates computed with constant and **b** time varying

winds, **c** SAM-index, **d** the difference between **e** SP anomalies in the eastern Weddell Sea and **f** SP anomalies in the eastern Bellingshausen Sea, as well as anomalies in the zonal position of the Amundsen Sea Low. Blue dots in all panels indicate months of enhanced SFR events identified in the time series shown in **a**, green dashed lines indicate the average over these months for the respective variable in each panel also stated as numeric value <SFR> together with standard deviation σ and linear trend including confidence intervals for each time series.

r	SFR	SFR*	SAM	EBS-EWS	EWS	EBS	ASL lon	ASL CP
SFR	1.00							
SFR*	**0.93**	1.00						
SAM	0.06	0.05	1.00					
EBS-EWS	**0.33**	**0.38**	**0.23**	1.00				
EWS	**-0.18**	**-0.23**	**-0.69**	**-0.55**	1.00			
EBS	**0.26**	**0.27**	**-0.27**	**0.76**	**0.12**	1.00		
ASL lon	**-0.15**	**-0.15**	0.02	**-0.41**	-0.03	**-0.52**	1.00	
ASL CP	0.07	0.09	**-0.60**	-0.05	**0.53**	**0.35**	**-0.14**	1.00

Supplementary Table 1. **Correlation coefficients between monthly anomalies.** spatially averaged (SFR) sea ice formation rates computed with constant and; (SFR*) time-varying winds; (SAM) Southern Annual Mode index; (EWS) pressure in the eastern Weddell Sea; (EBS) Eastern Bellingshausen Sea; (EBS-EWS) the pressure difference between these two locations, (ASL lon) the Amundsen Sea Low central longitude and; (ASL CP) the central absolute pressure. Bold numbers indicate statistical significance at $p = 0.01$.

r	SFR	SFR*	SIC	ΔT	U
SFR	1.00				
SFR*	**0.92**	1.00			
SIC	**-0.70**	**-0.57**	1.00		
ΔT	**0.14**	0.03	**0.13**	1.00	
U	**0.22**	**0.51**	**-0.13**	**-0.26**	1.00

Supplementary Table 2. **Correlation coefficients between monthly time series of spatially averaged anomalies of.** (SFR) sea ice formation rates computed with constant and; (SFR*) time-varying winds; (SIC) sea ice concentration; (ΔT) difference between the atmospheric temperature at 2 m height above the surface and the ocean surface freezing temperature and; (U) absolute wind speed at 10 m height above the surface. Bold numbers indicate statistical significance at $p = 0.01$.
